# Silicon Nanotubes Fabricated by Wet Chemical Etching of ZnO/Si Core–Shell Nanowires

**DOI:** 10.3390/nano10122535

**Published:** 2020-12-17

**Authors:** Yong-Lie Sun, Xiang-Dong Zheng, Wipakorn Jevasuwan, Naoki Fukata

**Affiliations:** 1 International Center for Materials Nanoarchitectonics, National Institute for Materials Science, 1-1 Namiki, Tsukuba 305-0044, Japan; zheng.xiangdong@outlook.com (X.-D.Z.); jevasuwan.wipakorn@nims.go.jp (W.J.); 2 Institute of Applied Physics, University of Tsukuba, 1-1-1 Tennodai, Tsukuba 305-8573, Japan

**Keywords:** silicon, nanotubes, core/shell nanowires, ZnO nanowire templates

## Abstract

Silicon nanotubes (SiNTs) have garnered a great deal of interest for both their synthesis and their potential for application to high-capacity energy storage, biosensors, and selective transport. In this study, we report a convenient and low-cost route to the fabrication of vertically aligned SiNTs via a wet-etching process that enables the control of the wall thickness of SiNTs by varying the gas flux and growth temperature. Transmission electron microscopy (TEM) characterization showed the resultant SiNTs to have an amorphous nature and a hexagonal hollow core. These SiNTs can be crystallized by thermal annealing.

## 1. Introduction

Nanotubular structures are characterized by a large specific surface area with more exposed active sites, giving them unique physical and chemical properties. Silicon nanotubes (SiNTs) are a highly promising semiconductor material due to their potential application to energy storage, biosensors, and chemical transport. The axial hollow space within the SiNTs can offer improved electrochemical performance for lithium-ion batteries since it provides additional free space to accommodate the massive volume expansion that occurs during the lithiation/delithiation process, thus preventing the pulverization of silicon [1,2,3,4,5,6,7]. The large specific surface area of SiNTs also minimizes the lithium-ion diffusion length and increases the ion flux. In other applications, biomolecular species can directly flow into the inner cavity to gate a SiNT transistor for application as an intracellular sensor [8]. The inner and outer surfaces of nanotubular structures can also be functionalized differentially to enable selective transport, separation, and filtering [9,10].

Today, SiNTs are mainly synthesized using 1-D sacrificial templates. A Si shell layer is deposited on a template such as ZnO [2,5], Ge [9,11], or MgO [12] nanowires, followed by selectively etching away the templates to form the SiNTs. Top-down techniques using lithography [13,14,15] and self-assembled nanosphere bead templates [16,17,18] have more recently been developed to fabricate SiNTs, but they require a complex manufacturing process, long processing time, or the use of noble metals. Other methods such as chemical etching [19] and electrochemical formation [20] cannot produce vertically aligned SiNTs, so they have limited applications.

Sacrificial templating methods based on ZnO nanowires (NWs) are regarded as a convenient, low-cost, and controllable method of fabricating SiNTs. Vertically aligned ZnO nanowires are synthesized using the hydrothermal method at temperatures of under 100 °C, followed by the deposition of a Si shell layer on the ZnO template using chemical vapor deposition (CVD). Finally, the ZnO core is selectively etched via a reduction process at 600 °C with 50% H_2_ in N_2_ for 24 h [2]. However, this etching process is highly energy and time consuming, hindering the future large-scale production of SiNTs.

In this study, we propose the use of a wet chemical etching method to remove the ZnO template instead of the gas-phase etching process, resulting in a convenient, cheap, and timesaving approach to the fabrication of vertically aligned SiNTs. We investigated the Si shell growth rate as a function of the precursor gas flux and growth temperature to control the SiNTs’ wall thickness. The morphology, crystallinity, and elemental composition of the SiNTs were characterized. We also studied the crystallization of Si shell layers with different shell thickness by thermal annealing.

## 2. Materials and Methods

An n-type Si(100) substrate was sputtered with 100 nm of ZnO to serve as a seed layer for nanowire growth. The growth solution consisted of 10 mM zinc nitrate, 5 mM hexamethylenetetramine (HMTA), and 0.6 M ammonium hydroxide. A small amount of sodium citrate (0.08 mM) was added to the solution to maintain the hexagonal cross-sectional shape of the nanowires. The substrate was then floated on the solution surface with its front side downwards and placed in an oven at 95 °C for 6 h to perform the hydrothermal growth. This growth condition was optimized to produce ZnO NWs with a large wire-to-wire spacing and high aspect ratio. All the chemicals were purchased from Wako Pure Chemical Industries (Tokyo, Japan).

After rinsing with deionized water and isopropyl alcohol (IPA), the samples were loaded into an ultra-high-vacuum chemical vapor deposition (UHV-CVD) chamber with a background pressure of 2 × 10^−6^ Pa. The Si shell layer was coated on the ZnO NWs using SiH_4_ as the precursor gas at temperatures of 650 and 700 °C. The growth time was increased from 6 to 20 min. The details of the UHV-CVD method are reported elsewhere [21,22,23]. The SiH_4_ gas flow was varied from 6 to 20 sccm, and the total pressure was set at 700 Pa by mixing with nitrogen gas.

**Figure 1:**
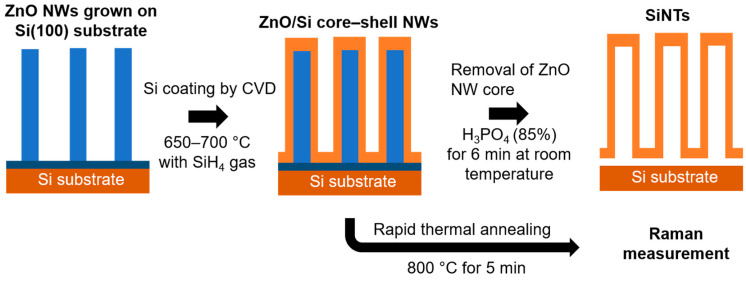
Schematic illustration of the fabrication procedure for silicon nanotubes (SiNTs).

To finally grow the Si nanotube structures, the ZnO cores were selectively wet-etched in phosphoric acid (85%) for 6 min at room temperature and then rinsed repeatedly with deionized water to remove any residual acid. Additionally, the ZnO/Si core–shell NWs were treated by rapid thermal annealing at 800 °C for 5 min under an N_2_ atmosphere to study the crystallization of the Si shell layers. The full fabrication procedure is illustrated in Figure 1.

Sample Characterization. Scanning electron microscopy (SEM) images were recorded using a Hitachi S-8000 SEM (Tokyo, Japan) at an acceleration voltage of 5 kV. Transmission electron microscopic (TEM) and energy-dispersive X-ray spectrometry (EDX) analyses were performed using a JEOL 2100F transmission electron microscope (Tokyo, Japan) at 200 kV. Micro-Raman scattering (Photon Design, Tokyo, Japan) measurements were carried out using a 355 nm excitation beam with a 100× objective, with the power set at 0.02 mW to prevent local heating effects [24,25]. X-ray diffraction (XRD) patterns were obtained using a PANalytical X’Pert Pro MRD system (Tokyo, Japan) with a parallel Cu Kα beam.

## 3. Results and Discussion

**Figure 2:**
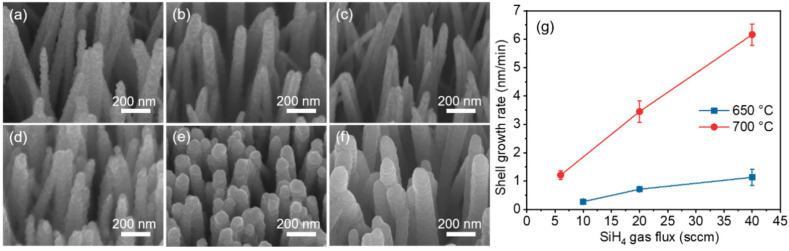
(**a**–**c**) 30°-tilted SEM images of vertically aligned ZnO NWs with a Si shell layer grown at 650 °C using 10, 20, and 40 sccm of SiH_4_ gas flux, respectively. (**d**–**f**) 30°-tilted SEM images of ZnO NWs with Si shell grown at 700 °C using 6, 20, and 40 sccm of SiH_4_ gas flux, respectively. (**g**) Growth rate as a function of the SiH_4_ gas flux at different growth temperatures. The growth time was varied for each growth condition to ensure an appropriate diameter.

Figure 2 shows scanning electron microscopy (SEM) images of the ZnO/Si core–shell NWs as a function of the Si shell growth temperature and SiH_4_ gas flux. The CVD process forms Si shell layers on the ZnO core, with thicknesses that vary from 12 to 21 nm. The Si shell growth rate increased sublinearly with SiH_4_ gas flux, which was caused by the higher density of the available source materials for its formation. Raising the growth temperature from 650 to 700 °C enhanced the Si shell growth rate due to the faster decomposition rate for the precursor gas at the higher temperature. To produce an appropriate shell thickness for each growth condition, the growth times for the samples shown in Figure 2a–f were set at 20, 20, 10, 15, 6, and 5 min, respectively.

**Figure 3:**
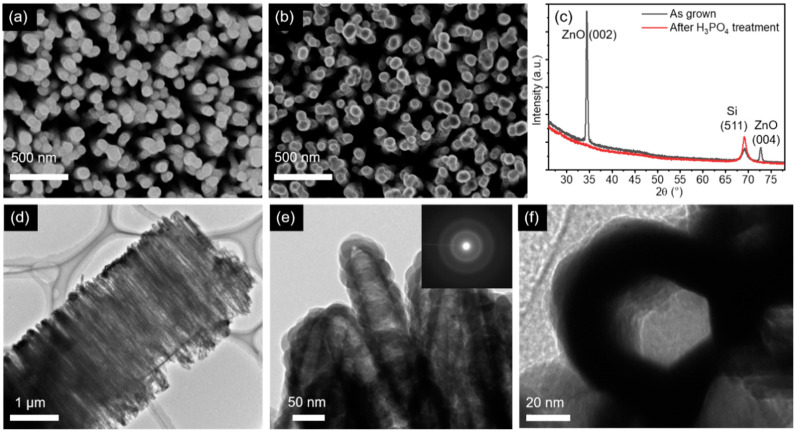
Top-view SEM images of ZnO/Si core–shell NWs (**a**) before and (**b**) after the wet-etching process. (**c**) Corresponding XRD pattern before and after the wet-etching process. (**d**) Low- and (**e**) high-magnification TEM images of the SiNTs. Corresponding selected area electron diffraction (SAED) pattern in the inset of (**e**) showing its amorphous nature. (**f**) Cross-sectional TEM image showing the hexagonal hollow space of the SiNTs.

The SEM and TEM images of the nanowires with Si shells grown at 700 °C using 20 sccm of SiH_4_ gas flux for 6 min are shown in Figure 3. The SEM image in Figure 3b shows a darker area at the center of every single nanowire compared to Figure 3a, indicating the formation of cavities after the wet-etching process. The XRD pattern recorded from the nanowires in Figure 3c shows an absence of ZnO peaks after etching, which confirms the removal of the ZnO core. The presence of the silicon peak is mainly due to the Si substrate, since the crystallinity of the Si shell is not perfect due to the growth on the ZnO surface being closer to amorphous. This amorphous nature will be also discussed later.

**Figure 4:**
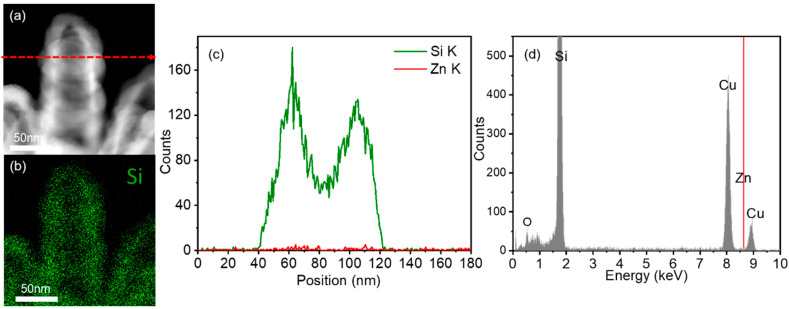
(**a**) TEM image and (**b**) EDX mapping for Si of the SiNTs. (**c**) EDX linescan for Si and Zn along the radial direction of a SiNT. The linescan position is shown as the dashed line in (**a**). (**d**) TEM-EDX spectrum recorded from the SiNTs, showing the absence of a Zn peak.

The TEM images in Figure 3d,e demonstrate the sealed tubular structure with a wall thickness of approximately 20 nm. The hazy halo pattern of the selected area electron diffraction (SAED) image in Figure 3e reveals the amorphous nature of the Si shell layer. The cross-sectional TEM image through the bottom of a nanotube in Figure 3f shows a hexagonal hollow space, which originates from the cross-sectional shape of ZnO nanowires. The elemental mapping and linescan analysis of the SiNTs are shown in Figure 4. The energy-dispersive X-ray (EDX) mapping and linescan for Si demonstrate the successful synthesis of SiNTs, and the TEM-EDX spectrum in Figure 4d shows that the ZnO core was completely etched out. Note that the Cu peaks in the spectrum come from the Cu grid. Moreover, the caps of these vertically aligned SiNTs can be etched using reactive-ion etching (RIE) [6] to produce open-cap SiNTs that have a wider potential range of applications.

**Figure 5:**
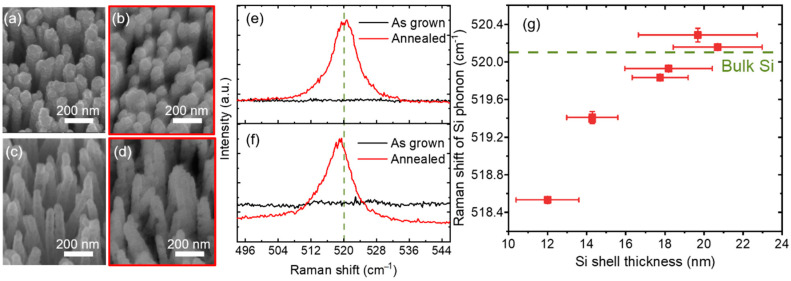
30°-tilted SEM images of ZnO/Si core–shell NWs with a thick (20 nm) shell layer (**a**) before and (**b**) after thermal annealing at 800 °C for 5 min. SEM images of the nanowires with a thin (12 nm) shell layer (**c**) before and (**d**) after thermal annealing. Corresponding Raman spectra for the nanowires with (**e**) thick and (**f**) thin shell layers. The green dashed line shows the bulk value. (**g**) Raman shift of Si optical phonon peak for nanowires with different shell thicknesses after annealing. The error bar indicates the standard deviation of the shell thickness and Raman measurements.

To improve the electrical and optical properties, ZnO/Si core–shell NWs were thermally annealed to crystallize the silicon shell layers. Figure 5a–d show almost no change in the morphology of the nanowires after thermal annealing at 800 °C for 5 min. Figure 5e,f show the results of Raman measurements at an excitation wavelength of 355 nm. This wavelength was selected to shorten the penetration depth in Si, resulting in the suppression of the signal from the ZnO core. No Raman peaks were observed before annealing for both samples with thick (20 nm) or thin (12 nm) Si shell layers, showing that the Si shell layers are not crystalline but amorphous. After annealing, the Si optical phonon peak shifted to higher wavenumbers and reached the bulk value (520.1 cm^−1^) upon increasing the Si shell thickness. The downshift and asymmetric broadening to lower wavenumbers observed for core–shell NWs with thin shell layers can be explained by the phonon confinement effect [24,26,27,28,29].

**Figure 6:**
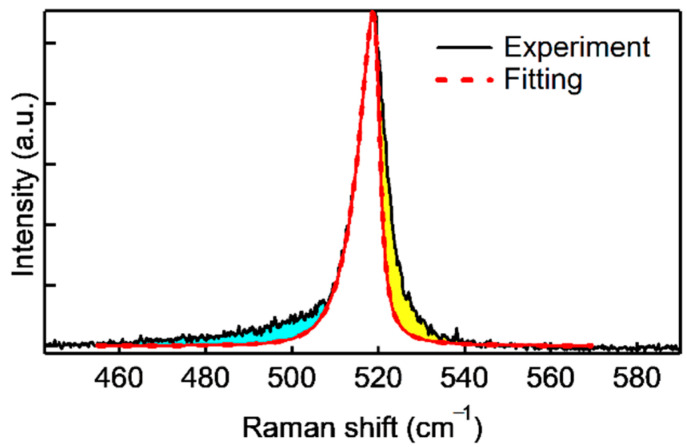
Si optical phonon peak observed for ZnO/Si core–shell NWs with a thin (~12 nm) Si shell layer. The dashed line indicates the fitting result using phonon confinement theory.

Richter et al. [26] and Campbell and Fauchet [27] reported that the phonon confinement model and the Raman intensity is given by

(1) $$I\left(\omega \right)=\int \frac{{\left|C\left(0,q\right)\right|}^{2}}{{\left[\omega -\omega \left(q\right)\right]}^{2}+{\left(\Gamma_{0}/2\right)}^{2}}d^{3}q$$

where *C*(0,*q*) is a Fourier coefficient of the confinement function, *ω*(*q*) is the Si phonon dispersion, and *Γ*_0_ is the full width at half maximum of the reference Si. Here, we used the following relations: ∣*C*(0*,q*)∣^2^ = exp(−*q*^2^*d*^2^/16*π*^2^) and *ω*(*q*) = [*A* + *B*cos(*qπ*/2)]^0.5^ + *D*, with *A* = 1.714 × 10^15^ cm^−2^ and *B* = 10^5^ cm^−2^ [25]. *D* is an adjusting parameter for the bulk Si. These relations can be used for a cylindrical structure such as an NW. The Si shell structure after removing the ZnO core is an NT structure and is different from a typical NW structure. Considering the small diameter of the Si/ZnO core–shell NWs, we treated the Si shell structures as pseudo-NW structures. The fitting result is shown in Figure 6. The phonon correlation length estimated by the fitting was 7–8 nm. This value is close to the thickness of the Si shell layers. The fitting is, however, not so good on the high- and low-wavenumber sides. There are several possible reasons for this. First, the relationship used for the approximation is that employed for NWs. Second, there is the problem of crystallinity. This is considered the main reason for the incompleteness of the lower-wavenumber fitting. Third, the effect of the strain from the ZnO forming the heterojunction, mainly due to the incompleteness of the fitting on the higher-wavenumber side, may be a factor. The Si optical phonon peak did, in fact, show a higher Raman shift than the value obtained for bulk Si (520.1 cm^−1^), as shown in Figure 5g. The upshift means that compressive stress is induced from the ZnO core region.

This is expected to be a promising approach to the development of battery materials that require low-cost and high-capacity materials. Our method is applicable to materials that can be selectively etched. For example, by replacing ZnO with a material such as Ge [22,23], epitaxial growth on Ge nanowires is possible: it is expected that nanotube structures with a smoother surface can be formed. Impurity doping is also important in making materials funcstional [29,30,31]. Since this CVD method can also be used for doping impurities, multiple future applications are anticipated.

In summary, we have developed a convenient and low-cost route to fabricating vertically aligned SiNTs by applying a wet-etching process to the ZnO region of ZnO/Si core–shell NWs. The Si shell growth rate can be controlled by adjusting the precursor gas flux and growth temperature. The fabricated SiNTs showed an amorphous nature, with hexagonal hollow spaces, and were crystallized by thermal annealing, showing that the crystallinity can also be controlled.
